# Feasibility study of functional near-infrared spectroscopy in the ventral visual pathway for real-life applications

**DOI:** 10.1117/1.NPh.11.1.015002

**Published:** 2024-01-08

**Authors:** Weilu Chai, Peiming Zhang, Xiaoyan Zhang, Jia Wu, Chao Chen, Fu Li, Xuemei Xie, Guangming Shi, Jimin Liang, Chaozhe Zhu, Minghao Dong

**Affiliations:** a Xidian University, School of Life Science and Technology, Engineering Research Center of Molecular and Neuro Imaging of Ministry of Education, Xi’an, China; b Xidian University, School of Life Science and Technology, Xi’an Key Laboratory of Intelligent Sensing and Regulation of trans-Scale Life Information, Xi’an, China; c Xidian University, School of Artificial Intelligence, Key Laboratory of Intelligent Perception and Image Understanding of Ministry of Education, Xi’an, China; d Northwestern Polytechnical University, School of Foreign Languages, Xi’an, China; e PLA Funding Payment Center, Beijing, China; f Xidian University, School of Electronics and Engineering, Key Laboratory of Intelligent Perception and Image Understanding of Ministry of Education, Xi’an, China; g Beijing Normal University, State Key Laboratory of Cognitive Neuroscience and Learning, Beijing, China

**Keywords:** fNIRS, feasibility, lateral occipital complex, fusiform face area, ventral visual pathway

## Abstract

**Significance:**

fNIRS-based neuroenhancement depends on the feasible detection of hemodynamic responses in target brain regions. Using the lateral occipital complex (LOC) and the fusiform face area (FFA) in the ventral visual pathway as neurofeedback targets boosts performance in visual recognition. However, the feasibility of utilizing fNIRS to detect LOC and FFA activity in adults remains to be validated as the depth of these regions may exceed the detection limit of fNIRS.

**Aim:**

This study aims to investigate the feasibility of using fNIRS to measure hemodynamic responses in the ventral visual pathway, specifically in the LOC and FFA, in adults.

**Approach:**

We recorded the hemodynamic activities of the LOC and FFA regions in 35 subjects using a portable eight-channel fNIRS instrument. A standard one-back object and face recognition task was employed to elicit selective brain responses in the LOC and FFA regions. The placement of fNIRS optodes for LOC and FFA detection was guided by our group’s transcranial brain atlas (TBA).

**Results:**

Our findings revealed selective activation of the LOC target channel (CH2) in response to objects, whereas the FFA target channel (CH7) did not exhibit selective activation in response to faces.

**Conclusions:**

Our findings indicate that, although fNIRS detection has limitations in capturing FFA activity, the LOC region emerges as a viable target for fNIRS-based detection. Furthermore, our results advocate for the adoption of the TBA-based method for setting the LOC target channel, offering a promising solution for optrode placement. This feasibility study stands as the inaugural validation of fNIRS for detecting cortical activity in the ventral visual pathway, underscoring its ecological validity. We suggest that our findings establish a pivotal technical groundwork for prospective real-life applications of fNIRS-based research.

## 1 Introduction

Functional near-infrared spectroscopy (fNIRS) is a promising non-invasive neuroimaging technique known for its portability, cost-effectiveness, and high ecological validity, making it particularly well-suited for neurofeedback studies conducted in real-life settings.^1^^,^^2^ In the field of neuroenhancement, fNIRS holds promise for improving visual recognition performance by targeting specific brain regions, such as the lateral occipital complex (LOC) and the fusiform face area (FFA) within the ventral visual pathway. However, despite an extensive literature review, the feasibility of utilizing fNIRS to detect activity in the LOC and FFA regions of adult participants remains insufficiently validated.

Several recent studies in the field of fNIRS have reported cortical activations within the ventral visual pathway, shedding light on the potential of this technique.^3^^–^^6^ For instance, Emberson et al. successfully identified LOC activation in infants, in which target channel locations were determined based on magnetic resonance imaging (MRI) data.^7^ Shimada employed brain coordinates from meta-analysis and a three-dimensional (3D) digitizer to pinpoint activation foci in the extrastriate body area in adults.^8^ Similarly, Hu et al. observed activations of occipitotemporal cortex in Chinese character recognition, identifying target cortices based on a 3D digitizer and off-line spatial registration.^9^ However, no existing literature definitively demonstrates the feasibility of utilizing fNIRS to measure hemodynamic responses in the LOC and the FFA among adult subjects. Thus, the feasibility of employing fNIRS to explore these specific regions in adults remains an unresolved matter.

**Fig. 1:**
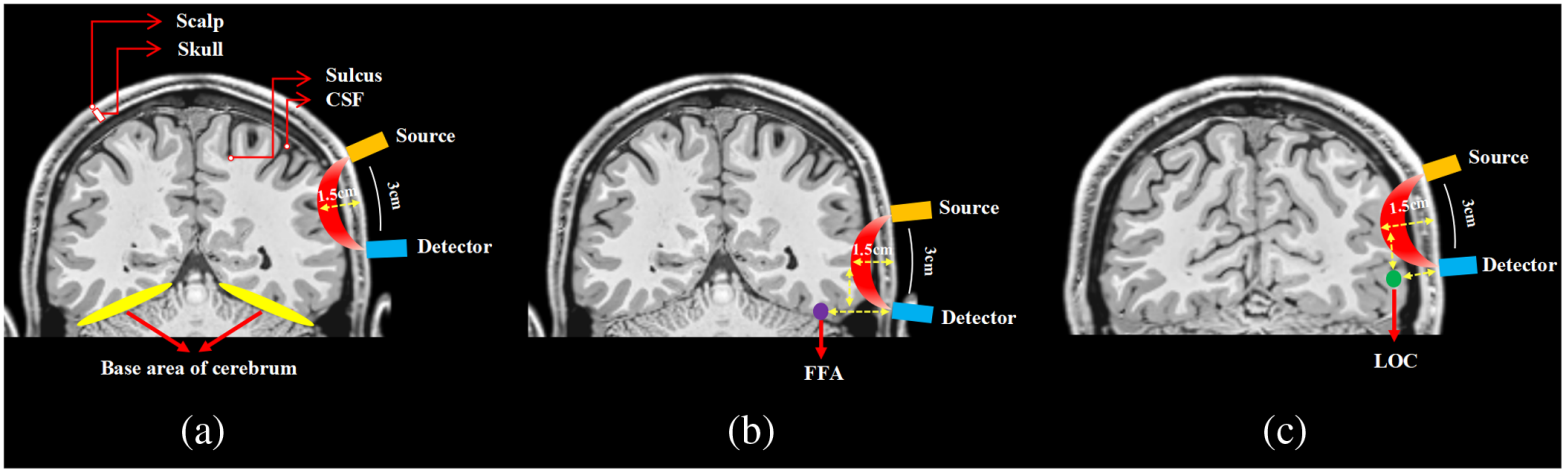
Illustration of the fNIRS detection range for cortical regions. (a) The principle of fNIRS detection is depicted, showing the path of fNIRS photons (red) traveling through the scalp, skull, and cerebrospinal fluid to reach the cerebral cortex. The fNIRS detection range is ∼1.5  cm, which corresponds to approximately half of the source–detector separation.^10^^,^^11^ (b) The spatial relationship between the fNIRS detection range and the center of FFA is illustrated. The center of FFA (MNI coordinate: 40, −52, −22^12^), indicated in purple, is likely to exceed the range of fNIRS detection. (c) The spatial relationship between the fNIRS detection range and the center of LOC is illustrated. The center of LOC (MNI coordinate: 46.58, −67.15, −6.24^13^), shown in green, is unlikely to exceed the range of fNIRS detection. The visualization of this figure was performed using MRIcron version 1 software.

As an optical brain imaging technique, fNIRS measures changes in brain tissue concentrations of oxygenated and deoxygenated hemoglobin following neuronal activation within the near-infrared range. The depth of detection with fNIRS is limited to ∼15 to 17.5 mm beneath the human scalp due to the scattering and absorption of near-infrared spectroscopy light through the brain tissues.^10^^,^^11^ Consequently, cortical areas located deep within the base of the cerebrum, such as the fusiform gyrus and the inferior temporal gyrus within the ventral visual pathway, as well as areas folded within the brain sulcus and the subcortical regions, are likely to exceed the detection limit of fNIRS [Fig. 1(a)]. Anatomically, the LOC resides in the lateral bank of the fusiform gyrus, encompassing the posterior inferior temporal gyrus and extending into the fusiform gyrus and the occipitotemporal sulcus.^14^^–^^17^ Similarly, the FFA, situated in the base of the cerebrum, is in close proximity to the anterior part of the fusiform gyrus.^18^^–^^22^ Given the anatomical positions of the LOC and FFA, detecting the hemodynamic responses in these regions poses a challenge for fNIRS as the depth of these regions may exceed the detection limit [as shown in Figs. 1(b) and 1(c)]. Specifically, we hypothesize that the FFA may be less readily detected by fNIRS than LOC due to its location potentially surpassing the detection threshold of fNIRS.

In this study, we aim to investigate the feasibility of utilizing fNIRS to measure hemodynamic responses in the ventral visual pathway, specifically targeting the LOC and the FFA, among adult participants. We recorded the hemodynamic activities of the LOC and the FFA in 35 subjects using a portable eight-channel fNIRS instrument. To accurately determine the placement of fNIRS optodes for both regions, we employed the transcranial brain atlas (TBA) developed by our research group.^23^^,^^24^ This approach allowed for precise spatial localization of brain regions on the scalp surface using the group Montreal Neurological Institute (MNI) coordinates of given regions, eliminating the need for individual structural MRI data. Brain responses in the LOC^25^ and the FFA^21^ were elicited using static two-dimensional images of daily objects and faces embedded in the one-back paradigm. This paradigm has been reliably employed in classical MRI studies to elicit activation in the LOC and FFA when compared to control conditions.^13^^,^^19^^,^^26^^,^^27^ Data analysis of the fNIRS recordings was conducted using the general linear model. To the best of our knowledge, this study represents the first investigation into the feasibility of fNIRS in measuring cortical activity within the ventral visual pathway. Our findings contribute to a deeper understanding of the capability and validity of fNIRS in real-life applications for measuring cortical activities within the ventral visual pathway.

## 2 Materials and Methods

### 2.1 Subjects

Thirty-five college students (25 males and 10 females) from a local university participated in this study. The participants had an average age of 23.77±2.36 years (mean ± standard deviation). All subjects were right-handed, as determined by the Edinburgh Handedness Questionnaire,^28^ and had normal or corrected-to-normal vision.

This study was approved by the Ethical Committee of Xidian University and was conducted in accordance with the Declaration of Helsinki. All participants were informed about the purpose and safety of the experiment, and written informed consent was obtained.

### 2.2 Stimuli and Experimental Design

The experiment was conducted in a quiet and dimly lit room. Participants were seated comfortably in a chair, facing a monitor placed on a desk. Visual stimuli were presented on the monitor, and participant responses were collected using a keyboard. Throughout the experiment, participants were instructed to remain calm and focused, minimizing head and body movements except when providing keystroke responses. The experimental task was implemented using the E-Prime program (version 3.0, Psychology Software Tools).

**Fig. 2:**
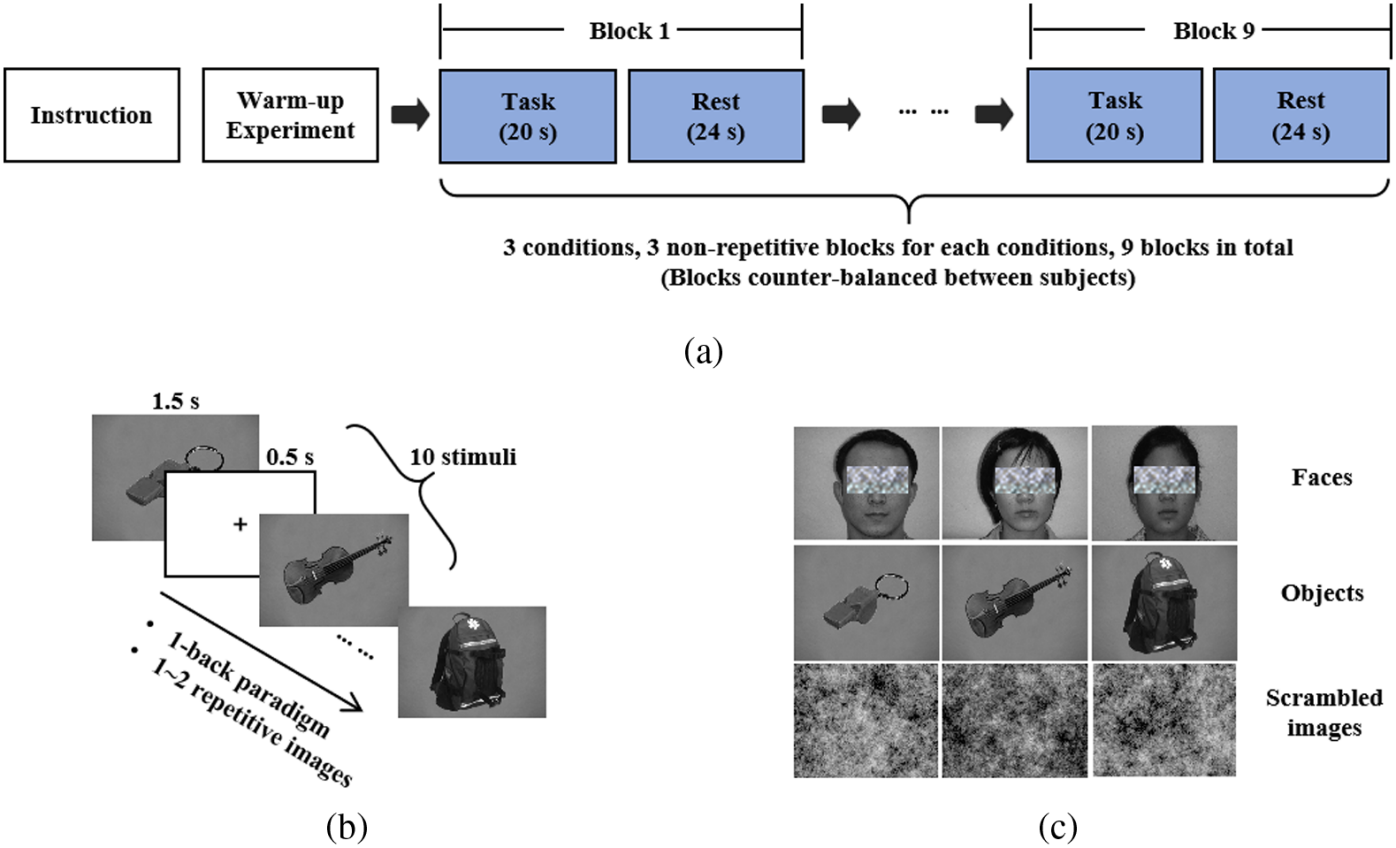
Illustration of stimuli and experimental design. (a) Experimental procedure: Following instructions and warm-up trials, the experiment consisted of nine blocks, with three blocks for each condition. Each block included a 20-s task period and a 24-s rest period. (b) Task period procedure: the task period employed a 1-back paradigm with 10 stimuli, including one to two repeated images. Different categories of stimuli were used in the task periods of the three condition blocks. (c) Stimuli from three different image libraries. The experiment employed stimuli from three categories: faces, objects, and scrambled images.

In this study, we employed the 1-back recognition task, which is commonly used as the standard experimental paradigm in fMRI studies to locate the LOC^25^ and the FFA.^21^ The experiment began with a series of warm-up trials to familiarize participants with the recognition task procedure. Subsequently, participants performed the recognition task under three conditions: objects, faces, and scrambled images [Fig. 2(a)]. Each condition consisted of three blocks, resulting in a total of nine blocks, which were presented in a random order to reach a counterbalance across participants. Each block included two periods: a 20-s task period and a 24-s rest period. During the task period, 10 stimuli were randomly presented from the corresponding image library, with 1 or 2 stimuli repeated. Each stimulus was displayed 1500 ms, followed by a 500 ms cross fixation [Fig. 2(b)]. In the task period, participants were instructed to press the number “1” key on the keyboard if they detected a repeated stimulus; otherwise, no response was required. During the rest period, a center fixation cross was displayed against a gray background, and participants were instructed to maintain fixation and clear their minds. The stimuli used in each condition were selected from three different image libraries [Fig. 2(c)]. The object image library comprised 20 images of everyday objects, such as bags, food, hats, and various tools. The face image library consisted of the face images of the 40 students from Xidian University without any background. The scrambled image library included 20 white noise images with various shapes. All image stimuli from the three libraries had a pixel size of 640×480.

### 2.3 fNIRS Data Acquisition

The fNIRS signals were recorded during the formal experiment using a portable fNIRS device (OctaMon+, Artinis Medical Systems, the Netherlands) with a sampling rate of 10 Hz, which has been widely employed in previous studies.^29^^–^^31^ This portable fNIRS device contains eight channels, including eight light-emitting diodes and two receivers operating at wavelengths of 760 and 850 nm. Based on the modified Beer–Lambert Law,^32^ the fNIRS device converts the light intensity signal into the oxyhemoglobin and deoxyhemoglobin concentration. Oxyhemoglobin is a more commonly used biomarker of cerebral activity due to its larger amplitudes compared with deoxyhemoglobin.^33^^–^^35^ Note that, in this study, we focused on analyzing the oxyhemoglobin concentration as the biomarker, and unless otherwise specified, all results presented in this paper are derived from the oxyhemoglobin concentration signals.

#### 2.3.1 fNIRS 3D localization method

In this study, we employed a precise 3D localization system, called TPen, based on the TBA to accurately determine the optimal placement of fNIRS optodes for the LOC and FFA.^23^^,^^24^ The TBA is a high-resolution and large-sample probabilistic mapping that accurately maps scalp locations to corresponding brain areas, providing reliable guidance for the placement of transcranial devices on the scalp surface.^23^ The TBA-based TPen system, which has been successfully implemented in previous fNIRS experiments such as finger-tapping and working memory studies, ensures accuracy, consistency, and efficiency in transcranial research.^23^^,^^24^

**Fig. 3:**
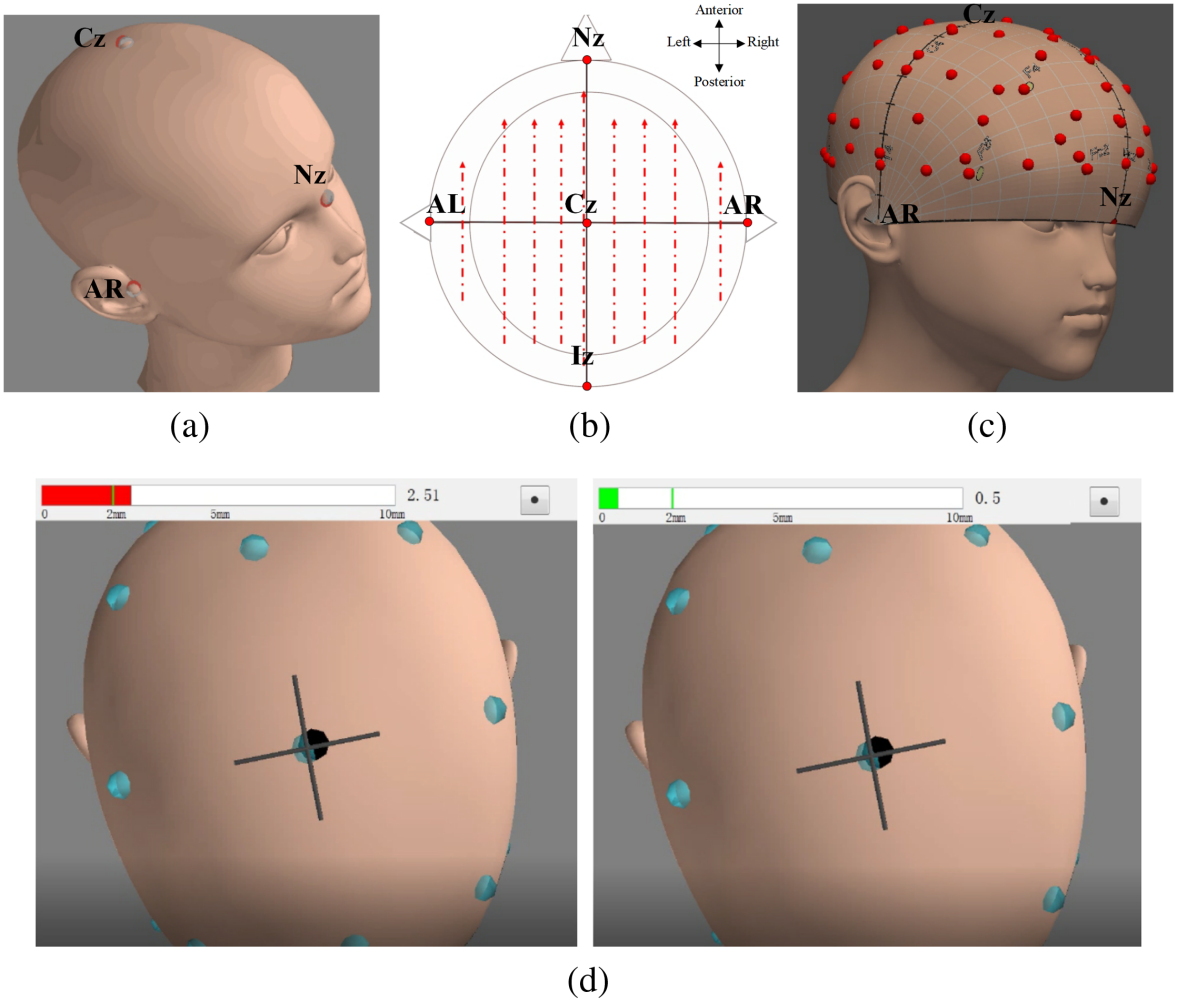
Process of localization utilizing the TPen system. (a) The first step is to establish five main reference points in the order specified by the system: Cz, Iz (inion), Nz (nasion), AR (anterior right ear), and AL (anterior left ear). (b) Subsequently, a series of uniformly distributed points are marked across the scalp along the approximate trajectory of the red line. (c) Finally, a CPC model is reconstructed by the system based on these marked sampling points. (d) The diagram illustrates the procedure of CPC coordinate navigation following the reconstruction of the CPC model. The CPC coordinates are input as navigation target points (gray dots). As the locator probe pen traverses over the subject’s head, its position is displayed in real time (black dot) to correspond with its location. The red bar located at the top of the figure indicates the distance between the black and gray target dots. When the black dot approaches the target gray point <2 mm, the red bar turns green, indicating that the location is within an acceptable margin of error from the target.

In our study, we utilized a 3D magnetic digitizer (Polhemus) in the TPen system to reconstruct a 3D model of each subject’s scalp. This model enabled us to create a continuous proportional coordinates (CPC) spatial representation specific to each individual. Using this CPC model, we visually and dynamically positioned the fNIRS probes, ensuring precise placement of the LOC and FFA channels based on their respective CPC coordinates with an error margin of <2  mm. The CPC coordinates were derived by converting the MNI coordinates of the most activated voxels in previous studies focusing on the LOC and FFA, utilizing the TPen system. Figure 3 provides a visual representation of the localization process using the TPen system.

#### 2.3.2 Target channels configuration

In our study, we recognize that LOC and FFA are not individual voxels but rather clusters. However, each fNIRS channel position corresponds to a single pixel coordinate in CPC space, located at the center of the detector and receiver. When multiple channels are established through multi-point sampling of LOC or FFA, it becomes challenging to arrange them on the corresponding small area of the scalp due to the required 2 to 3 cm interval between fNIRS channels. Therefore, we selected the most activated voxel from both LOC and FFA as our target channels.

**Fig. 4:**
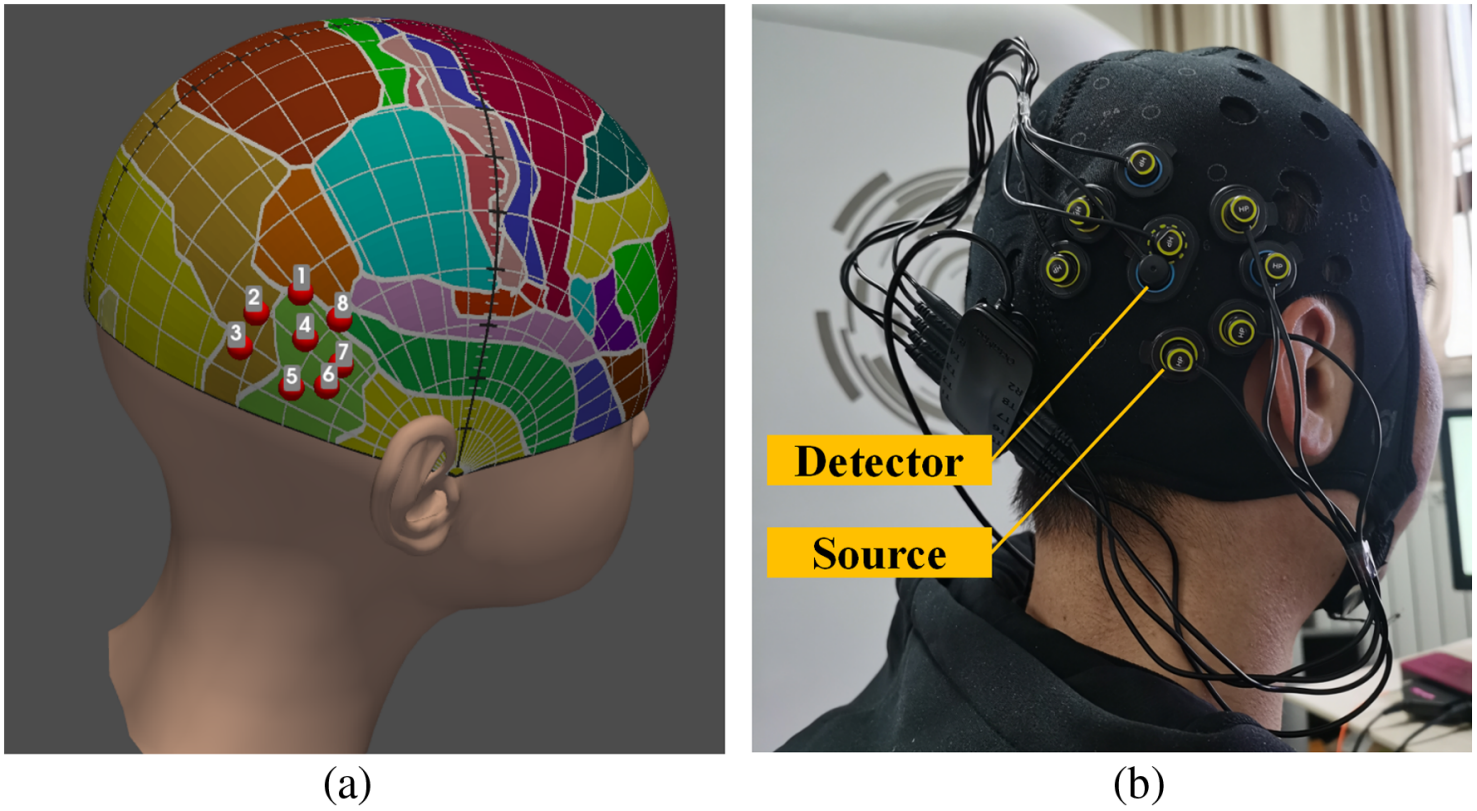
3D localization and configuration for target channels. (a) The configuration for all channels. The red dot represents the location of the channel, and the number on it indicates its corresponding label. The LOC target channel is CH2 (shown in red dot 2) and the FFA target channel is CH7 (shown in red dot 7). (b) The 8×1 probe arrangement (consisting of eight sources and one detector) for each subject.

For the current study, we selected the most activated voxels of right-LOC (Talairach coordinates: 42, −64, −6.7,^13^ converted to MNI coordinates:^36^ 46.58, −67.15, −6.24) and the right-FFA (MNI coordinates: 40, = −52, −22^12^) from previous studies as our two target coordinates. These two target coordinates were then converted into the CPC coordinates (CPC of right-FFA: 0.90, 0.82; CPC of right-LOC: 0.86, 0.76).^23^^,^^24^ Using the TPen system, the 3D CPC model for each subject was constructed. Subsequently, the two target CPC coordinates were located on the CPC model as the LOC and FFA target channels (CH2 and CH7, respectively, shown in Fig. 4). The adjacent channels were positioned in close proximity to the target channels, with CH1 and CH3 located near the LOC target channel (CH2) and CH5, CH6, and CH8 situated close to the FFA target channel (CH7), as shown in Fig. 4(a). To alleviate the influence of skin blood flow, a linear regression reference channel was established with a short source–detector separation distance (<1  cm),^37^ as shown in Fig. 4(a). Finally, the 8 × 1 probe arrangement (consisting of eight sources and one detector) for each subject is shown in Fig. 4(b); it comprises eight channels including two target channels (CH2, CH7), adjacent channels (CH1, CH3, CH5, CH6, CH8), and a short channel (CH4).

### 2.4 fNIRS Data Analysis

#### 2.4.1 fNIRS data preprocessing

To preprocess and analyze the fNIRS data, we employed Matlab R2021b along with NIRS_KIT (version 1.3).^38^ The preprocessing steps were performed to enhance the quality of the data and mitigate various sources of noise and artifacts.

First, we removed the initial 20 s, which served as a warm-up period, to eliminate any potential instabilities present at the beginning of the recording. To mitigate the influence of skin blood flow, the linear regression was conducted using a reference channel with a short source–detector separation distance.^37^ In addition, a linear detrending procedure was applied to remove slow drifts caused by temperature changes in the portable fNIRS equipment and subtle head movements of the subjects. Addressing motion-related artifacts, we utilized the temporal derivative distribution repair technique.^39^ This method effectively corrected for any head and body movement noise, resulting in cleaner data for further analysis. Furthermore, to minimize the influence of physiological artifacts, including cardiac pulsation (1 Hz),^40^ respiratory signal (about 0.3 Hz),^40^^,^^41^ and blood pressure (Mayer wave) oscillation (0.08 to 0.12 Hz),^40^ we implemented a bandpass filter with a range of 0.01 to 0.08 Hz (IIR, third order). This filtering step attenuated these artifacts while preserving the experimental frequency (∼0.023  Hz).

#### 2.4.2 Statistical analysis

In our study, we performed a comprehensive statistical analysis to examine the hemodynamic responses under different experimental conditions.

First, at the individual level, we applied a generalized linear model based on the canonical hemodynamic response function (HRF)^42^ to estimate the hemodynamic response and generate HRF fit weight coefficients (β values) for the preprocessed fNIRS data in the target and adjacent channels. Moving to the group-level analysis, we conducted a normality test to assess the distribution of β values within each group. This step aided in selecting an appropriate method for significance testing. Subsequently, we performed significance tests to compare the hemodynamic responses across different conditions, and sensitivity analysis was carried out to determine the effect size (Cohen’s *d*)^43^ of the observed differences between the groups. In addition, we calculated the average hemodynamic response signal curves in the target and adjacent channels for the three conditions across subjects. These curves were compared to the canonical HRF for reference.

Through these rigorous statistical analyses, we aimed to identify significant differences in hemodynamic responses across experimental conditions. The consideration of effect sizes allowed us to assess the practical significance of these differences. This comprehensive analysis provided valuable insights into the impact of different experimental conditions on the measured hemodynamic responses using fNIRS data.

## 3 Results

### 3.1 Results of Behavioral Task

To assess the subjects’ attention and engagement during the experiment, we collected and calculated the keystroke accuracy from the 1-back recognition task. The behavioral results demonstrated that the participants performed at a consistently high level, with an average accuracy of 98.97±0.56% (mean ± SD). Although the task itself was relatively simple, the high average accuracy suggests that the subjects maintained a strong level of concentration throughout the experiment. This finding supports the notion that the participants were actively focused and attentive during the task, reinforcing the reliability of the collected data.

### 3.2 Results of fNIRS Data Analysis

Selective hemodynamic responses in the LOC and FFA channels were examined through a group-level analysis employing significance tests and sensitivity analysis. A normality test conducted on the β values revealed significant deviations from the normal distribution. Therefore, the paired Wilcoxon sign rank test, a nonparametric test, was chosen for the subsequent significance analysis. This test was applied to assess significant differences in hemodynamic responses of all channels under three conditions, involving a total of 35 subjects. In addition, a sensitivity analysis was performed to quantify the magnitude of significant differences between groups, considering the current sample size.

#### 3.2.1 Results of hemodynamic responses in LOC

**Fig. 5:**
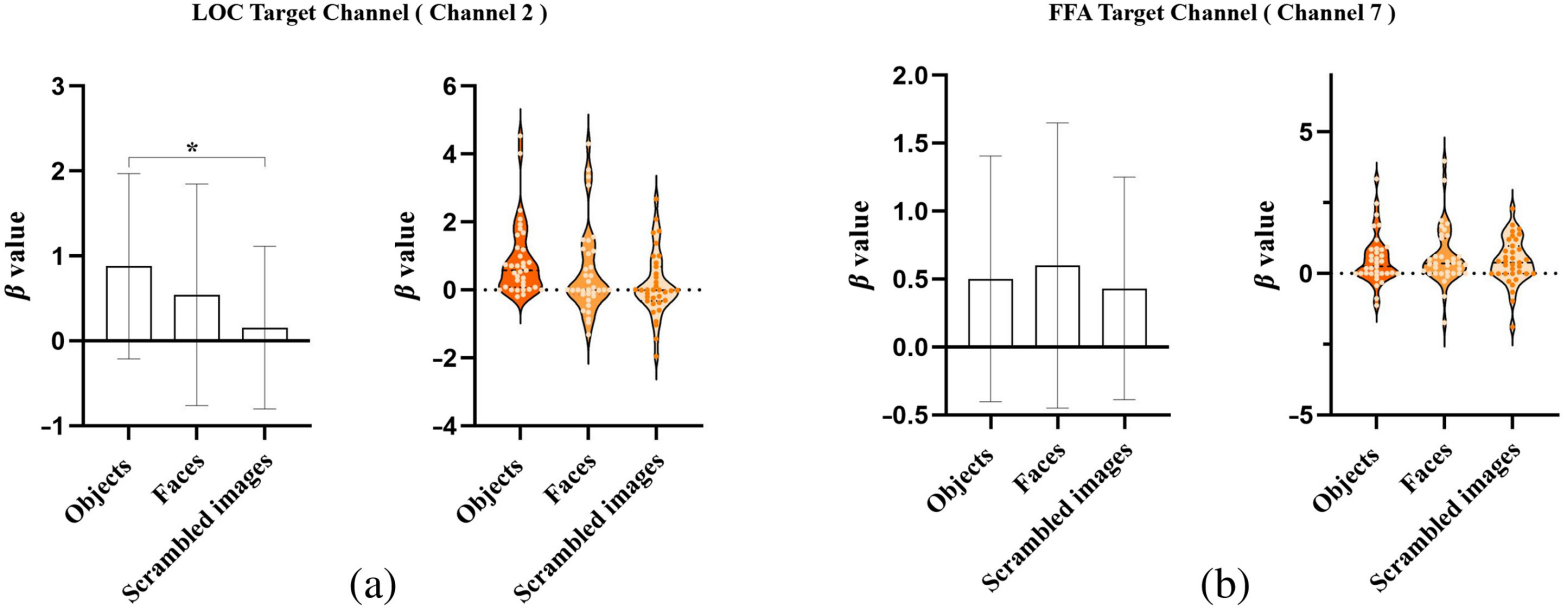
Hemodynamic differences between conditions in two target channels (n=35). (a) Hemodynamic differences in the LOC target channel shown by the bar plot and the violin plot. The β values under the objects condition were significantly greater than those under the scrambled images condition. (b) Hemodynamic differences in the FFA target channel shown by the bar plot and the violin plot. The β values under the faces condition were not significantly greater than under the objects condition. Note: * indicates the significant differences between groups (p<0.05).

The results of hemodynamic responses in the LOC target channel (CH2) are shown in Fig. 5(a) and Table 1. The HRF fit weight coefficients (β values) for the objects condition were significantly higher than those for the scrambled images condition (p=2.7031e-7<0.05,z=5.143). The sensitivity analysis in the LOC target channel is illustrated in Table 1. With a sample size of 35, the effect size of the significant difference between the objects and scrambled images conditions was found to be sufficiently large (Cohen’s d=0.7049),^44^ indicating significantly greater hemodynamic responses in the LOC target channel for objects compared with scrambled images. Outlier detection was performed, and the exclusion of outliers did not alter the results of hemodynamic responses in the LOC target channel. Moreover, the hemodynamic responses in adjacent channels (CH1, CH3) of the LOC target channel (CH2) did not exhibit significant differences under both object and scrambled image conditions, as demonstrated in Table 1 and Figs. S1(a) and S1(b) in the Supplementary Material.

**Table 1:** Results of paired Wilcoxon sign rank test and sensitivity analysis.

Channel	Contrast	Paired Wilcoxon sign rank test		Sensitivity analysis
		p value	Z value	Effect size (Cohen’s d)
CH1	Object – scrambled	0.9869	0.0163	0.1189
CH2(LOC)		2.7031e-7	5.1430	0.7049
CH3		0.0797	1.7525	0.1926
CH5	Face–object	0.3421	0.9499	0.2702
CH6		0.8186	0.2293	0.0074
CH7(FFA)		0.5123	0.6551	0.1007
CH8		0.9217	0.0982	0.1083

#### 3.2.2 Results of hemodynamic responses in FFA

The results of hemodynamic responses in the FFA target channel (CH7) are presented in Fig. 5(b) and Table 1. The β values of the faces condition did not show a significant difference compared with the objects condition (p=0.5123>0.05, z=0.6551). The sensitivity analysis in the FFA target channel is provided in Table 1. With a sample size of 35, the effect size of the difference between the faces and objects conditions was small (Cohen’s d=0.1007),^44^ indicating that the hemodynamic responses in the FFA target channel did not exhibit a significant difference between faces and objects. Outlier detection was conducted, and excluding the outliers did not affect the results of hemodynamic responses in the FFA target channel. Additionally, the hemodynamic responses in adjacent channels (CH5, CH6, CH8) of the FFA target channel (CH7) also did not demonstrate significant differences under both faces and object image conditions, as demonstrated in Table 1 and Figs. S1(c)–S1(e) in the Supplementary Material.

#### 3.2.3 Results of average hemodynamic response curves

**Fig. 6:**
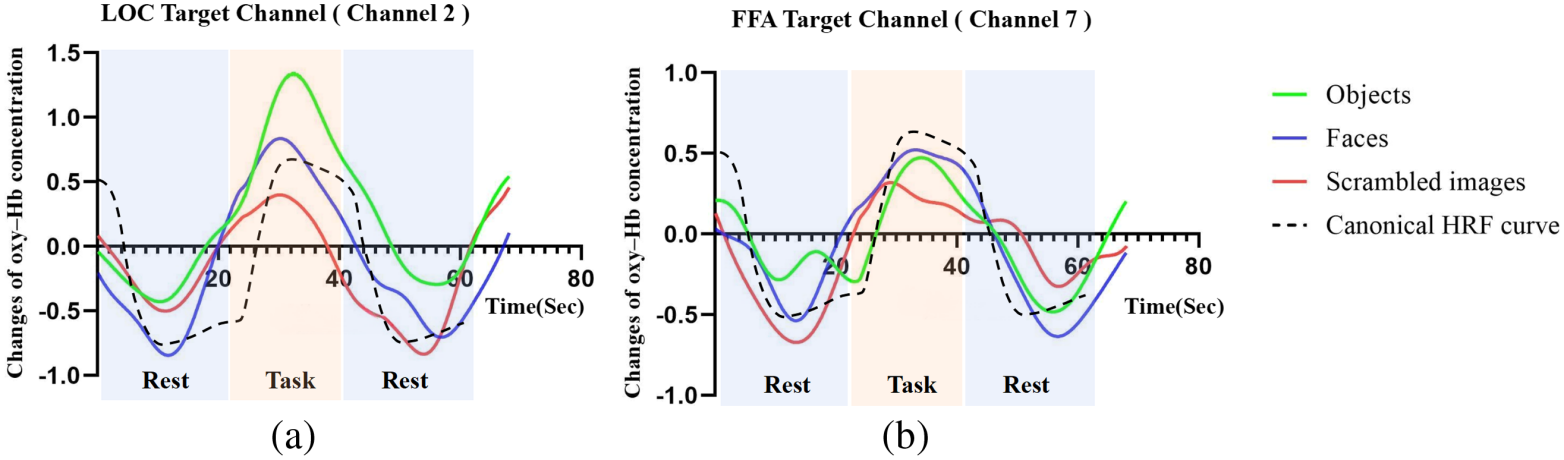
Average hemodynamic response curves in two target channels (n=35). (a) Average hemodynamic response curves in LOC target channel under three conditions. (b) Average hemodynamic response curves in FFA target channel under three conditions.

The signals from all subjects (n=35) under three conditions were averaged, and the results are depicted in Fig. 6. The average hemodynamic response curve for the objects condition in LOC target channels (CH2) exhibited higher oxyhemoglobin concentration and better canonical HRF fitness compared with the curves for the faces and scrambled images conditions [shown in Fig. 6(a)]. Similarly, the average hemodynamic response curve for the faces condition in FFA target channels (CH7) displayed a slightly higher oxyhemoglobin than the curves for the objects and scrambled images conditions, although the differences were not substantial [shown in Fig. 6(b)]. In addition, Fig. S2 in the Supplementary Material illustrates the average hemodynamic response curves under three conditions in adjacent channels of the LOC and FFA target channels. Similar to the FFA target channel, these curves in each adjacent channel did not exhibit notable differences between the three conditions.

## 4 Discussion

The present study aims to investigate the feasibility of using fNIRS to measure hemodynamic responses in the ventral visual pathway, specifically targeting the LOC and FFA regions in adults. Our results demonstrated that the LOC target channel (CH2) exhibited selective hemodynamic responses in response to object recognition tasks as evidenced by significantly greater HRF fit weights for the objects condition compared with the scrambled images condition [p<0.05, z=5.143, as shown in Fig. 5(a) and Table 1]. On the other hand, the FFA target channel (CH7) did not show selective hemodynamic responses to faces under the stimulus contrast of faces versus objects as there was no significant difference in HRF fit weights between the faces and objects conditions [p>0.05, z=0.6551, as shown in Fig. 5(b) and Table 1]. In comparison with the selective hemodynamic responses observed in previous fMRI studies investigating the LOC and FFA regions, our findings suggest that fNIRS is a promising tool for detecting hemodynamic responses in the LOC but may have limitations in the FFA. Our findings provide valuable insights into the capability and limitations of fNIRS for detecting cortical activity in these regions, contributing to the understanding of fNIRS-based neuroenhancement and its potential real-life applications.

### 4.1 Relevance of This Study for Real-Life Applications

The relevance of this study for real-life applications lies in its contribution to addressing key challenges associated with using fNIRS in real-world scenarios. fNIRS is a promising neuroimaging technique due to its non-invasive nature, portability, and cost-effectiveness, making it suitable for real-world application research. However, there are two critical challenges that need to be addressed: the feasible detection of hemodynamic responses in target brain regions and finding a balance between localization accuracy and portability in the choice of localization methods.

The study provides supportive evidence in both of these aspects. In terms of the feasible detection of the ventral visual pathway, the study demonstrates the feasibility of using fNIRS to measure the LOC within the ventral visual pathway. This finding is significant as the anatomical position of this pathway has posed a challenge for fNIRS. The technical pipeline established in the study can also be applied to explore the feasibility of assessing other cortical regions within the ventral neural pathway using fNIRS, such as the extrastriate body area, the parahippocampal place area, and the visual word form area, which are also located in the base of the cerebrum.

Regarding the challenge of balancing accuracy and portability in localization methods, the study evaluates different localization methods and highlights the suitability of the TBA-based TPen system for real-world fNIRS applications. The traditional fNIRS localization method relies on the 10-20 system, which has limitations in terms of localization resolution. Consequently, researchers can only infer the location of functional brain regions based on the positions of the activation channels.^45^^,^^46^ Currently, several accurate 3D localization methods are known; these include fMRI co-registration, 3D magnetic space digitizer, and the TBA-based TPen system employed in this study. The fMRI co-registration localization method involves attaching an imaging substance, such as vitamin E, to the fNIRS probe location, followed by multiple fMRI scans and co-registrations.^47^^,^^48^ Although this method offers high resolution and accuracy, it demands highly cooperative subjects and is impractical in real-world experimental settings due to its complexity. Another 3D localization method, the 3D magnetic spatial digitizer, simplifies the process of localizing fNIRS channels but requires offline spatial registration and cannot achieve real-time localization.^8^^,^^9^ In comparison, the TBA-based TPen system allows real-time and accurate positioning for each individual, making it the most suitable localization method for real-world fNIRS applications. Thus, this study selected and verified the reliability of the TBA-based TPen system, highlighting its suitability for real-world fNIRS applications.

Overall, this study contributes to the advancement of fNIRS as a neuroimaging tool for real-life applications by addressing challenges related to the feasible detection of target brain regions and the choice of localization methods. It provides a foundation for further research in using fNIRS in real-world scenarios.

### 4.2 Feasibility of fNIRS for Measuring LOC

The signal measured by fNIRS, similar to fMRI, captures the hemodynamic response in the brain associated with changes in cerebral blood flow and volume.^49^ Experimental designs involving the same paradigm and stimuli can activate a consistent hemodynamic response pattern. In line with classical fMRI studies that localize the LOC, our study employed the same experimental design by comparing the hemodynamic responses of the LOC with object images and scrambled images.^14^^,^^16^^,^^25^^,^^26^^,^^50^^,^^51^ These classical fMRI studies consistently reported significant hemodynamic responses in the LOC when subjects observed object images compared with scrambled images.^14^^,^^16^^,^^26^^,^^50^^,^^51^ This hemodynamic response characteristic of the LOC is attributed to its selectivity for physical shapes.^15^^,^^52^

The results of our study successfully replicated this shape-selective characteristic of the LOC. Specifically, the HRF fit weight coefficients (β values) of the LOC target channel (CH2) under the objects condition were significantly greater than under the scrambled images condition [shown in Fig. 5(a) and Table 1], with effect sizes meeting expectations (sufficiently large, shown in Table 1).^44^ These findings indicate that the hemodynamic response in the LOC target channel was stronger when subjects viewed daily object images compared with scrambled images, aligning with the conclusions of classical fMRI studies on localizing the LOC.

Note that 6 out of 35 subjects (17.14%) had beta values close to zero for all conditions in the LOC target channel (CH2), suggesting that their CH2 signal quality may be poor [as shown in Fig. 5(a)]. We propose that the poor signal quality in these subjects is likely to be attributed to factors such as head movement, thick hair, or individual differences in cranial morphology.^3^^,^^5^^,^^7^^,^^50^^,^^53^^–^^57^ Addressing poor signal quality in participants has long been a challenging issue in studies employing fNIRS. Previous studies on the LOC and FFA using fMRI have commonly excluded subjects with poor imaging quality due to excessive head motion or insignificant activation, with proportions ranging from 7.69% to 22.5% of the total sample.^50^^,^^53^^–^^56^ Similarly, in previous fNIRS studies, subjects with poor signal quality were excluded, with percentages ranging from 13.33% to 33.33%.^3^^,^^5^^,^^7^^,^^57^ Although the exclusion of such subjects is common practice, we did not exclude them from the group analysis of all channels to maintain the rigor of the analysis. In future experiments, we plan to conduct fMRI verification to investigate the underlying factors contributing to these individual differences.

It is worth mentioning that CH1 and CH3 did not demonstrate significant object selectivity for LOC (objects versus scrambled images, CH1: p=0.9869>0.05, z=0.0163; CH3: p=0.0797>0.05, z=1.7525) in comparison with the significant object selectivity observed at CH2 (objects vs. scrambled images: p=2.7031e-7<0.05, z=5.143). Detailed information can be found in Table 1.

Considering all of the results collectively, our current study reaffirms our previous conclusion that the TBA method can accurately locate the target brain region.^23^^,^^24^ Moreover, our study provides valuable insight by demonstrating that placing the target channel corresponding to the CPC is sufficient for measuring LOC activity, negating the necessity for additional supplementary channels around the target CPC.

To the best of our knowledge, no previous fNIRS study has investigated shape selectivity in the adult LOC. Although previous fNIRS studies have successfully targeted the LOC in infants using precise localization methods (magnetic resonance localization), demonstrating selective responses to object shape,^7^ there have been no prior fNIRS studies replicating shape-selective activation in the LOC of adults.^6^^,^^8^^,^^9^^,^^47^ Therefore, our study represents the first evidence that accurately measures shape selectivity in the adult LOC using fNIRS.

### 4.3 Feasibility of fNIRS for Measuring FFA

The experimental design employed in our study to measure the FFA was based on the classical fMRI studies that aim to localize the FFA. These fMRI studies consistently reported a face-selective characteristic in the FFA, with stronger hemodynamic responses when subjects viewed face images compared with object images.^19^^,^^20^^,^^22^^,^^25^^,^^27^^,^^58^ However, our fNIRS results did not demonstrate a face-selective characteristic in the FFA target channel signal when subjects observed face images and object images. Specifically, the HRF fit weight coefficients of the FFA target channel under the face condition were not significantly larger than those under the object condition [shown in Fig. 5(b) and Table 1], and the effect sizes did not meet our expectations (shown in Table 1).^44^ In conclusion, our findings suggest that the fNIRS device used in this study was unable to measure the FFA in adults.

There are two likely reasons that the fNIRS device was unable to measure FFA in adults. The main reason is the limited penetration capability of fNIRS. Using different near-infrared wavelengths (650 and 925 nm), fNIRS devices can detect the change in the relative concentrations of light-absorbing rates for deoxyhemoglobin and oxyhemoglobin.^49^ Due to the NIR light scattering and light-absorbing, the NIR light attenuation occurs as it travels through the biological tissue, leading to the limited penetration capability of fNIRS (about 15 to 7.5 mm for adults).^10^ In addition, the anatomical location of the FFA may be beyond the reach of fNIRS. The FFA has an average size of $1\;{\mathrm{cm}}^{3}$,^27^ and its center MNI coordinates used in this study (40, −52, −22^12^) are ∼4.01  cm away from its corresponding CPC coordinates (0.90,0.82) on the scalp.^23^^,^^24^ Consequently, the limited penetration capability of fNIRS equipment is likely the primary reason for the inability to measure FFA in adults. The second possible reason is that the experiment design may have been insufficient for eliciting a hemodynamic response in FFA. However, in our study, the experiment design, including the paradigm and type of stimuli (shown in Fig. 2), was identical to that of classical fMRI studies that aimed to localize the FFA. Hence, the issue with measuring the FFA using the fNIRS device is unlikely to be caused by the experimental design. In conclusion, the most probable reason for the fNIRS device’s failure to measure the FFA in adults is the limited penetration capability of fNIRS.

In summary, this finding is consistent with our hypothesis that fNIRS detection in the FFA region may be limited, potentially due to its depth exceeding the detection threshold of fNIRS. These results highlight the challenges associated with detecting activity in deeper brain structures using fNIRS and emphasize the need for further investigation and refinement of fNIRS methodologies for targeting such regions.

## 5 Limitation

This study provides a technical foundation for future research; however, it is essential to consider several limitations when interpreting the results. First, the fNIRS instrument utilized in our study employed the standard two-wavelength near-infrared light commonly used in fNIRS research,^29^^–^^31^ with wavelengths of 760 and 850 nm. Although increasing the number of near-infrared light wavelengths potentially enhances signal estimation accuracy,^59^ it does not affect the inherent penetration capability of fNIRS. Consequently, augmenting the number of wavelengths is unlikely to alter the observed properties in our current findings. Nonetheless, we encourage future replication of our research using fNIRS instruments equipped with a greater number of wavelengths. Second, our study specifically examined the brain responses of two cortical regions, namely LOC and FFA, within the ventral visual pathway. Future investigations could explore the potential feasibility of employing fNIRS to measure other brain regions at varying depths within the ventral visual pathway, such as the extrastriate body area,^60^ the visual word form area,^61^ and the parahippocampal place area.^62^ Finally, our study provides further evidence that placing the target channel corresponding to the CPC is sufficient for measuring LOC activity, negating the necessity for additional supplementary channels around the target CPC. However, further research is needed to validate the generalizability and applicability of this insight to other target regions.

## 6 Conclusion

In summary, this study established the feasibility of utilizing fNIRS to measure brain responses in the LOC within the ventral visual pathway. Nevertheless, detecting responses in the FFA using fNIRS proved to be a challenging task. These results contribute significantly to our comprehension of both the capabilities and constraints of fNIRS in examining brain activity within the ventral visual pathway. Our study not only reinforces our previous conclusion^23^^,^^24^ but also provides significant insight by indicating that placing the target channel according to the target CPC is sufficient for measuring LOC activity, without the need for additional supplementary channels around the target CPC. In addition, this research offers a crucial technical foundation for future studies aiming to employ fNIRS in real-life scenarios, thereby advancing our exploration of brain activity in this pathway.

## Data Availability

The data that support the findings of this article are not publicly available due to ethical concerns. They can be requested from the author at weiluchai@stu.xidian.edu.cn.
