# Supplementary material for: Feasibility study of functional near-infrared spectroscopy in the ventral visual pathway for real-life applications
Source: Neurophotonics. 2024 Jan 8;11(1):015002. doi: 10.1117/1.NPh.11.1.015002 (PMC10773254; doi:10.1117/1.NPh.11.1.015002)
Supplement: Supplementary file 1 [file NPh_011_015002_SD001.pdf]

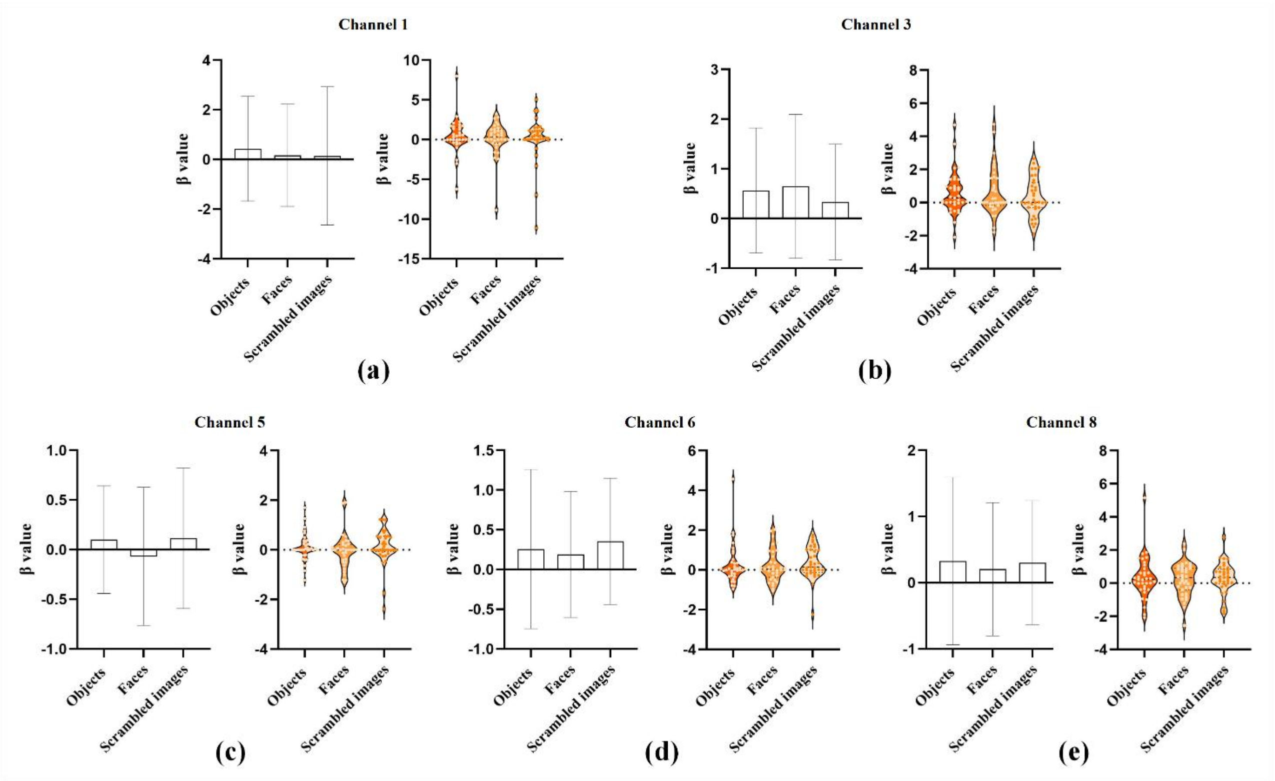

Supplementary Figure S1| Hemodynamic Differences in Adjacent Channels. (a) Bar plot and violin plot illustrating the hemodynamic differences in channel 1 (CH1), adjacent to the LOC target channel. The β values under the objects condition were not significantly greater than those under the scrambled images condition. (b) Bar plot and violin plot illustrating the hemodynamic differences in channel 3 (CH3), adjacent to the LOC target channel. The β values under the objects condition were not significantly greater than those under the scrambled images condition. (c) Bar plot and violin plot illustrating the hemodynamic differences in channel 5 (CH5), adjacent to the FFA target channel. The β values under the faces condition were not significantly greater than those under the objects condition. (d) Bar plot and violin plot illustrating the hemodynamic differences in channel 6 (CH6), adjacent to the FFA target channel. The β values under the faces condition were not significantly greater than those under the objects condition. (e) Bar plot and violin plot illustrating the hemodynamic differences in channel 8 (CH8), adjacent to the FFA target channel. The β values under the faces condition were not significantly greater than those under the objects condition.

Note: * Each plot represents the comparison of hemodynamic responses between different conditions in the respective adjacent channel. The absence of significant differences suggests that the adjacent channels did not exhibit the expected hemodynamic response patterns observed in the target channels.

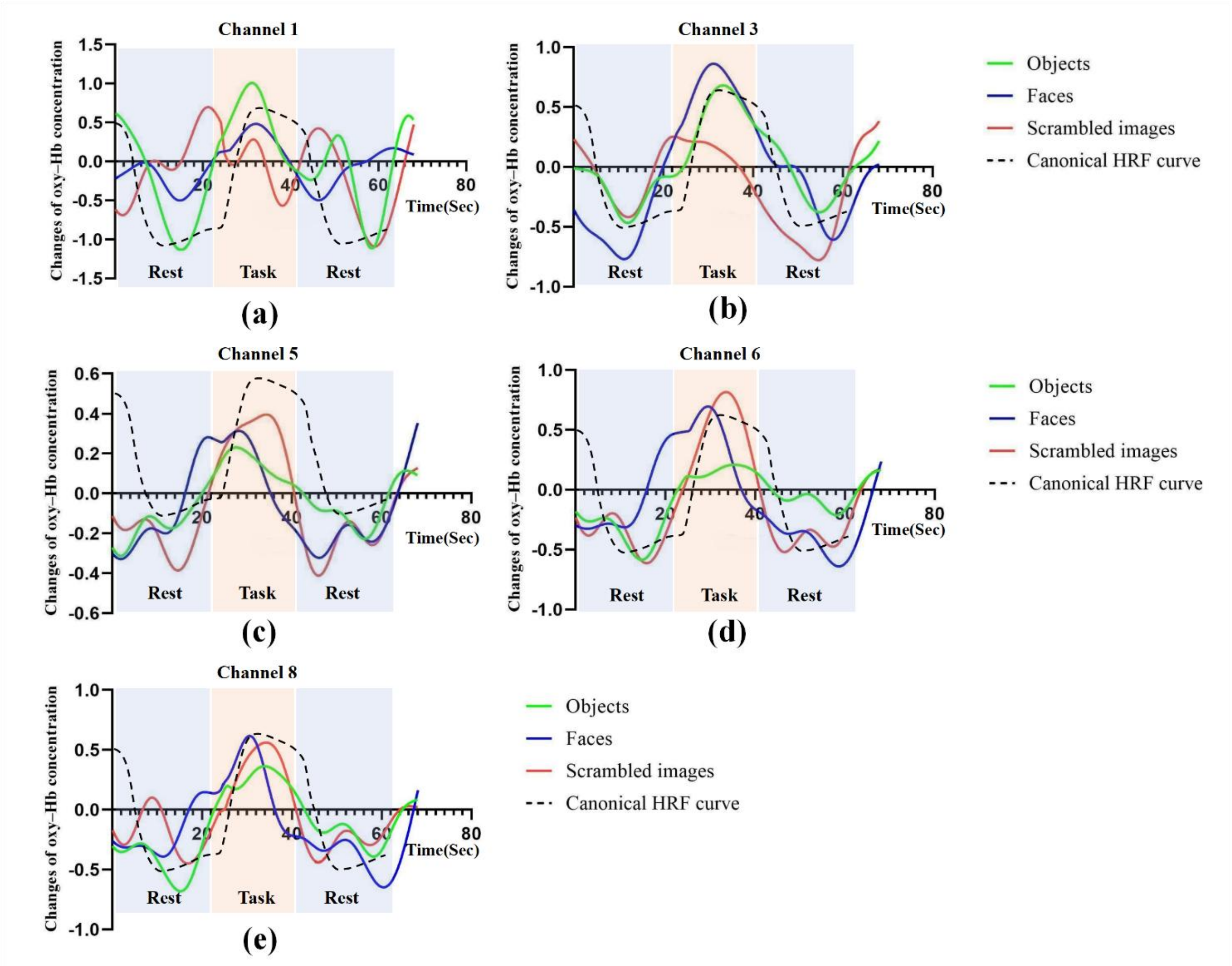

Supplementary Figure S2| Average hemodynamic response curves in adjacent channels (n=35). **(a)** Average hemodynamic response curves in channel 1 (CH1) adjacent to LOC target channel under three conditions. **(b)** Average hemodynamic response curves in channel 3 (CH3) adjacent to LOC target channel under three conditions. **(c)** Average hemodynamic response curves in channel 5 (CH5) adjacent to FFA target channel under three conditions. **(d)** Average hemodynamic response curves in channel 6 (CH6) adjacent to FFA target channel under three conditions. **(e)** Average hemodynamic response curves in channel 8 (CH8) adjacent to FFA target channel under three conditions.

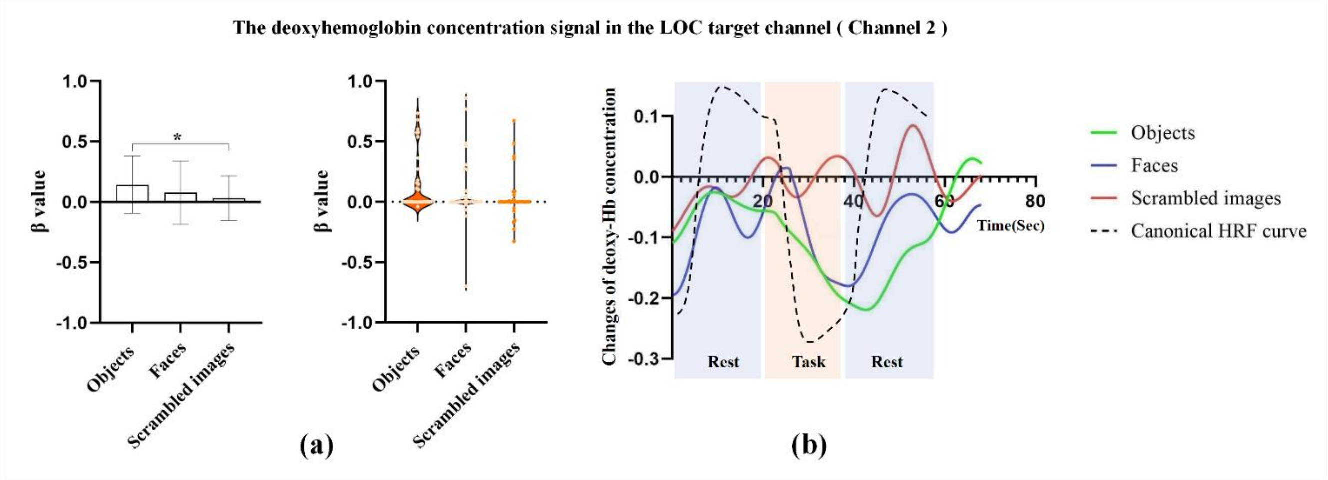

Supplementary Figure S3| The group-analysis results of the deoxyhemoglobin concentration signal in the LOC target channel (n=35). **(a)** Hemodynamic differences of the deoxyhemoglobin concentration signal in the LOC target channel were shown by the bar plot and the violin plot. The β values under the objects condition were significantly greater than under the scrambled images condition. **(b)** Average hemodynamic response curves of the deoxyhemoglobin concentration signal in the LOC target channel under three conditions. Note: * indicates the significant differences between groups ($p < 0.05$)
